# Initiation of anti-osteoporotic drugs in high-risk female patients starting glucocorticoid treatment: a population study in Norway

**DOI:** 10.1007/s11657-020-00783-8

**Published:** 2020-08-05

**Authors:** Ellen M. Apalset, Astrid Lunde, Mari Hoff, Vera Ehrenstein, Grethe S. Tell

**Affiliations:** 1 Department of Global Public Health and Primary Care, University of Bergen, Kalfarveien 31, N-5018 Bergen, Norway; 2 Bergen Group of Epidemiology and Biomarkers in Rheumatic Disease, Department of Rheumatology, Haukeland University Hospital, Bergen, Norway; 3 Department of Neuromedicine and Movement Science, NTNU – Norwegian University of Science and Technology, Trondheim, Norway; 4 Department of Public Health and Nursing, NTNU – Norwegian University of Science and Technology, Trondheim, Norway; 5 Department of Rheumatology, St. Olavs University Hospital, Trondheim, Norway; 6 Department of Clinical Epidemiology, Aarhus University, Aarhus, Denmark

**Keywords:** Glucocorticoids, Anti-osteoporotic drugs, Osteoporosis, Fracture, Inflammatory rheumatic diseases

## Abstract

***Summary*:**

Glucocorticoid use is a risk factor for osteoporosis and fractures. We studied whether women initiating glucocorticoid treatment also started anti-osteoporotic treatment, according to clinical guidelines. Women with versus without previous fracture were twice as likely to start anti-osteoporotic treatment within 1 year after initiating glucocorticoid treatment, but the cumulative incidences were low 9.1% vs. 4.6%, respectively.

**Purpose:**

Use of glucocorticoids (GC) is a risk factor for osteoporosis and fractures, and clinical guidelines suggest that preventive treatment with anti-osteoporotic drugs (AOD) should be considered when starting GC. Women with high risk of osteoporosis comprise those with previous fractures or a known inflammatory rheumatic disease, for whom the indication of AOD is even stronger. The purpose of these analyses was to investigate whether women initiating GC treatment also started AOD, especially those with high risk of osteoporosis.

**Methods:**

We used data from the Norwegian Prescription Database to identify all women 55 years and older initiating GC treatment in Norway during 2010–2016 and to obtain information on use of AOD. Data from the Norwegian Patient Registry were used to obtain information on previous fractures and diagnoses.

**Results:**

Among 105,477 women initiating GC treatment during 2010–2016, 3256 had started AOD and 79,638 had discontinued GC treatment after 1-year follow-up. Cumulative incidence of starting AOD after 1 year was 9.1% (95% CI: 7.9, 10.4) for women with vs. 4.6% (95% CI: 4.4%, 4.8%) for women without a previous fracture. Women with rheumatoid arthritis or another inflammatory rheumatic disease were more likely to start AOD than women with other indications. For the whole cohort, the probability of starting AOD treatment within 1 year after initiating GC increased on average 3% per year (HR = 1.03, CI: 1.01, 1.05) from 2010 to 2016.

**Conclusions:**

Having had a previous fracture or an inflammatory rheumatic disease increased the probability of treatment with AOD. However, the proportions starting AOD were much lower than clinically indicated.

**Electronic supplementary material:**

The online version of this article (10.1007/s11657-020-00783-8) contains supplementary material, which is available to authorized users.

## Introduction

Glucocorticoids (GCs) are part of the standard treatment in many conditions including inflammatory, autoimmune, and allergic diseases, cancer, and organ transplantations. Prescription of long-term therapy with GC (3 months or more) has been increasing, and around 1% of the population (1998–2008) of the UK were treated with GC [1]. Major side effects include osteoporosis and fractures. Use of GC is the most common cause of secondary osteoporosis [2, 3], and 30–40% of all patients treated with GC have radiological evidence of vertebral fractures [4, 5]. Risk of bone loss and fracture rises rapidly after GC treatment initiation [6–8]. In addition, for persons with similar bone mineral density (BMD), the risk of vertebral fractures is larger for GC users than for nonusers [9], indicating an additional effect on bone quality. The harmful effects on bone are dose-dependent, but no safe dose limit has been established [8].

According to both older and newer guidelines in Europe and the USA, the threshold for starting preventive treatment with anti-osteoporotic drugs (AOD) after initiation of GC medication should be low. This applies especially to high-risk patients (e.g., those with a history of low-energy fracture) or if GC therapy is intended to last for more than 3 months [10–12]. These recommendations were also implemented in the guidelines from the Norwegian Society of Rheumatology in 2015 [13]. However, overall use of AOD in Norway is lower than in other European countries [14–16]: in a Norwegian study, only 22.5% of women already receiving GC were treated with AOD during the first year after a forearm fracture [15]. Another Norwegian study found that use of AOD was highest in areas with the historically lowest incidence of osteoporotic fractures [17]. This indicates that, among those at highest risk of suffering a fracture, a suboptimal proportion receives anti-osteoporotic treatment.

GCs have for several decades been an important part of therapy in several inflammatory rheumatic diseases; patients with rheumatoid arthritis (RA), for example, may receive GC over many years [18, 19]. Treatment with GC may have a bone protective effect in highly inflammatory states [20], and the majority of newly diagnosed RA patients are treated with GC in the early stages to achieve rapid disease remission [21]. Both the disease and the treatment with GC are potentially harmful to the bone, and RA is the only medical condition considered to be a separate risk factor in the fracture risk assessment tool FRAX® [22]. There has been increasing emphasis on limiting use of GC to short periods and in moderate doses [23], but studies have not found a reduced use of GC in patients with RA [1, 19]. Another inflammatory rheumatic disease, giant cell arteritis (GCA), requires high doses and often long-term treatment with GC due to risk of serious complications including blindness if not adequately treated. Until recently, there has been no alternative treatment to GC for this condition, and in Norway, GC is still preferred due to low economical costs. Polymyalgia rheumatica (PMR), despite being commonly regarded as a disease with low morbidity, is one of the most common indications for starting GC [1]. Because PMR patients are often older than patients with RA at disease onset, they have a higher prevalence of previous fractures [24]. In addition, many of them sustain new fractures during GC treatment [24]. Even though the risk of osteoporosis and fractures due to long time GC therapy is substantial, the attentiveness to osteoporosis prophylaxis has been low [25].

Although treatment guidelines are clear and most physicians are aware of the detrimental effect of GC on bone, the degree to which these guidelines are adhered to in clinical practice is not clear. Thus, our primary aim was to examine the initiation of osteoporosis prophylaxis with AOD in women starting treatment with oral GC. We further investigated whether high-risk groups, i.e., women with a history of fracture or a rheumatic inflammatory disease, were more likely to receive AOD after starting GC. Finally, we examined changes in the prescribing rates of AOD at GC initiation from 2010 to 2016.

## Methods

### Study design and study population

This was a nationwide cohort study using data from the National Registry of Norway [26], the Norwegian Prescription Database (NorPD) [27, 28], the Norwegian Patient Registry (NPR) [29], the Norwegian Cause of Death Registry [30], and the Norwegian Education Database [31]. In Norway, every resident is assigned a unique identification number which enables exact linkage of each individual’s data from various registries. Government-funded hospitals and specialty clinics are required to report to the NPR, and diagnoses from all in- and outpatient hospital encounters have been recorded since 2008 [32]. The Norwegian Cause of Death Registry provides information on deaths and has high completeness [33]. The NorPD contains information on all prescription dispensings at outpatient pharmacies, ordered by physicians in both primary and specialist health care.

The source population consisted of 1,004,067 Norwegian women aged 55 years or older registered in the National Registry of Norway during 2010–2016. The study population consisted of all women initiating GC treatment (GC naïve), defined as having at least one GC dispensing from an outpatient pharmacy during the study period and with no dispensings during a 5-year look-back period. Prednisolone is the most commonly used GC outpatient treatment regimen, while prednisone is not a registered drug in Norway. Only systemic GC was studied (Table S1/Supplementary).

**Fig. 1:**
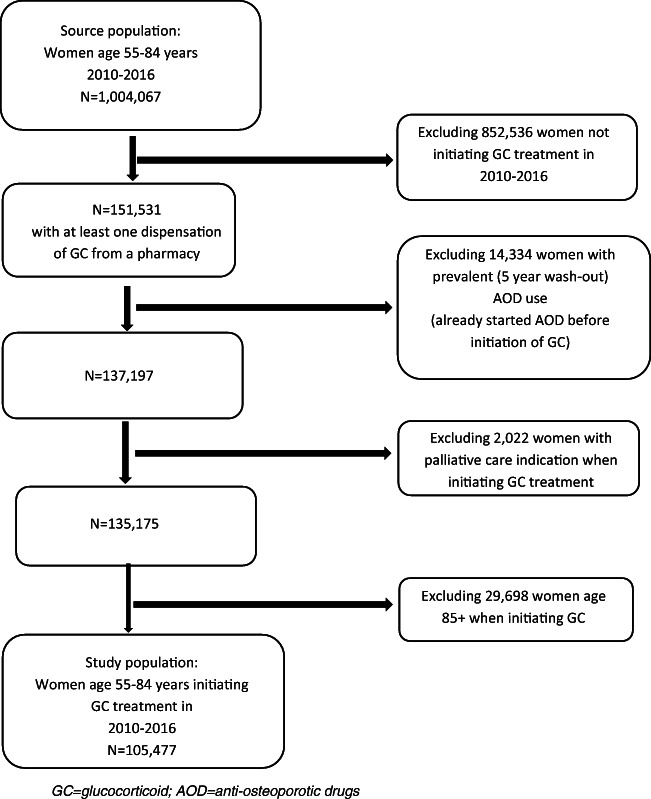
Norwegian women 55–84 years old initiating glucocorticoid (GC) treatment during 2010–2016. Flowchart of study population with inclusion and exclusion criteria

Only AOD naïve women initiating GC were included, excluding prevalent AOD users at the onset of GC treatment (5-year look-back) (Fig. 1). We expected a high proportion of women 85 years or older to live in nursing homes, and medication administered there are not registered in the NorPD; thus, women aged 85+ years at GC initiation treatment were excluded. Finally, women initiating GC treatment due to palliative care in cancer were excluded (identified through reimbursement codes, described below). The study population thus included 105,477 AOD naïve women (55–84 years) starting GC treatment, with follow-up through 2017.

### Time at risk

A patient was considered at risk of starting AOD treatment from the date of the first GC dispensing until discontinuation of GC treatment, the latter defined as a period without a new GC dispensing of 180 days or longer (grace period) beyond what was expected based on the days supplied in the last-recorded GC dispensing. Impact of the chosen grace period was examined in a sensitivity analysis where the grace period was changed to 90 days.

### Exposure

Fracture diagnoses for the hip, femur, ulna, radius, spine, pelvis, and humerus were obtained in the 2-year pre-GC baseline period and used as the exposure in the analyses (Table S2/Supplementary).

In Norway, GC treatment for certain diagnoses is reimbursed through governmental funding (i.e., at no, or low, cost for the patient). Such reimbursement diagnosis codes were used to identify the underlying disease (“GC indications,” Table S3). Reimbursement diagnosis code belonging to the first GC dispensing were categorized into four indication groups and used as exposure: “rheumatoid arthritis,” “other inflammatory rheumatic disease,” “other indications,” and “indication not known” (supplementary Table S3). Women without reimbursement at first GC dispensing (60%) or with reimbursement but with missing reimbursement diagnosis code (5%) were categorized according to the ICD-10 diagnoses that could be indications for GC treatment, obtained from the NPR during the 2-year baseline period. In case of more than one potential indication, the most recent diagnosis relative to the first GC dispensing was used. The category “other indications” includes chronic obstructive lung disease, asthma, sarcoidosis, dermatitis, and ulcerative colitis (Table S4/Supplementary).

### Outcome

The time from the first GC dispensing until the first dispensing of AOD was used as the outcome in the analyses, treating discontinuation of the GC treatment as a censoring event, death as a competing event (cumulative incidence) or censoring event (Cox regression), and emigration or end of the study period (31 December 2017), whichever came first, as censoring events. AOD included in the study are listed in Table S1 (Supplementary).

### Covariates

Diagnoses describing comorbidity were obtained according to the Romano modification of the Charlson comorbidity index (CCI) [29, 34]. This modification differs from the original CCI in that it includes dementia but excludes HIV. It further includes inflammatory rheumatic disease categories (connective tissue disease) such as rheumatoid arthritis, GCA, systemic lupus erythematosus (SLE), dermatopolymyositis, systemic sclerosis, and PMR. Information on relevant drug use (except GC and AOD) was collected for each woman in the 2-year baseline period preceding GC initiation. We did not adjust for CCI in the final analysis, as this only altered the associations slightly.

Education, age, and year at GC initiation were all adjusted for in the Cox regression analyses. Information on highest achieved education was obtained from the Norwegian Education Database [35] and categorized into basic (compulsory), secondary (high school/vocational education), or tertiary (college or university).

### Statistical analyses

Distributions of continuous variables were reported as medians and interquartile ranges; categorical variables were summarized using counts and percentages. Cumulative incidence of initiating AOD was calculated using the Aalen-Johansen method for competing risk, treating death as a competing event, stratified by having a history of fracture, among all women initiating GC and among women with known indication for GC treatment, stratified by GC indication, and stratified by Defined Daily Dose (DDD) of GC initially given (< 90 DDD and > = 90 DDD). Due to the low number of patients at risk after 5-year follow-up, we used 5 years as the maximum follow-up when calculating cumulative incidence.

Cox regression was used to estimate associations (HRs) between women with versus without a previous fracture and initiation of AOD at 1-year follow-up, stratified by GC indication and by DDD of GC initially given. In this analysis, death and termination of GC treatment were treated as censoring events. Analyses were adjusted for age at the index date (5-year age groups) and year initiating GC (linear term) and education. Due to few GC naïve patients with a previous fracture starting AOD treatment after 1-year GC treatment, we only present hazard ratios for fracture versus no previous fracture for starting AOD within 1 year after GC treatment and not for longer follow-up periods.

Age-standardized incidence rates of initiating AOD within 1 year from start of GC treatment were calculated using the direct method with 5-year age intervals using all women 55–84 years in Norway as the standard, treating discontinuation of GC treatment, death, and emigration as censoring events.

We calculated time trends of initiating AOD within 1 year of starting GC treatment, calculating hazard ratios using Cox regression, adjusting for age and education. We tested for possible interactions between time trend and GC indication and with previous fracture.

In all analyses, 95% confidence intervals (CIs) were calculated, and all analyses were conducted using R.

### Ethics

The study was approved by the Regional Committee for Medical and Health Research Ethics and by the Norwegian Data Protection Authority.

## Results

Baseline characteristics of the study population are presented according to a 2-year pre-baseline history of fracture (Table 1). About 3.7% of the study population had a fracture before initiation of GC, and the women in this group were older, and they had a higher comorbidity in most disease categories and a higher CCI score than women without fracture. Use of immunosuppressant medication other than GC was slightly higher for the fracture group. Women without a previous fracture had higher educational level than women with a fracture. While 3.0% in the total cohort had a diagnosis of rheumatoid arthritis registered in the NPR, 8.6% in the no fracture group and 8.8% in the previous fracture group had a rheumatoid arthritis reimbursement code from the primary or the specialist health care at the first GC dispensing.

**Table 1:** Baseline characteristics of Norwegian women (55–84 years) starting treatment with glucocorticoids (GC) in the period 2010–16, stratified by previous fracture registered in a 2-year look-back period before starting GC treatment (*N* = 105,477)

	Previous fracture (*N* = 3899) Number (%)	No previous fracture (*N* = 101,578) Number (%)
Median age (years) at initiation of GC (IQR)	70 (63,77)	67 (61,74)
Age at initiation of GC (*N*, %)		
55–64 years	1116 (28.6)	40,186 (39.6)
65–74 years	1402 (36.0)	37,391 (36.8)
75–84 years	1381 (35.4)	24,001 (23.6)
Year starting GC treatment (*N*, *%*)		
2010	499 (12.8)	13,672 (13.5)
2011	573 (14.7)	13,901 (13.7)
2012	593 (15.2)	14,901 (14.7)
2013	601 (15.4)	14,637 (14.4)
2014	560 (14.4)	15,176 (14.9)
2015	548 (14.1)	14,926 (14.7)
2016	525 (13.5)	14,365 (14.1)
Number of Defined Daily Doses dispensed of GC at initiation of GC (*N*, *%*)		
< 90 DDD	3519 (90.3)	92,368 (90.9)
> = 90 DDD	380 (9.7)	9210 (9.1)
Education (*N*, *%*)		
Basic (compulsory)	1320 (33.9)	32,487 (32.0)
Secondary (high school/vocational)	1877 (48.1)	48,500 (47.7)
Tertiary (college/university)	658 (16.9)	19,429 (19.1)
Unknown/missing	44 (1.1)	1162 (1.1)
CCI score (*N*, *%*)		
0	2292 (58.8)	71,907 (70.8)
1–2	932 (23.9)	20,279 (20.0)
3+	675 (17.3)	9392 (9.2)
Comorbidity categories^a^ (*N*, *%*)		
Myocardial infarction	240 (6.2)	3939 (3.9)
Congestive heart failure	187 (4.8)	2005 (2.0)
Peripheral vascular disease	132 (3.4)	2244 (2.2)
Cerebrovascular disease	222 (5.7)	2709 (2.7)
Dementia	68 (1.7)	390 (0.4)
Chronic pulmonary disease	654 (16.8)	9957 (9.8)
Connective tissue disease/inflammatory rheumatic disease^b^	217 (5.6)	4567 (4.5)
Rheumatoid arthritis	157 (4.0)	3033 (3.0)
Giant cell arteritis	14 (0.4)	235 (0.2)
Systemic lupus erythematosus	6 (0.2)	220 (0.2)
Dermatopolymyositis	1 (0.0)	56 (0.1)
Polymyalgia rheumatica	43 (1.1)	1088 (1.1)
Systemic sclerosis	6 (0.2)	96 (0.1)
Ulcer disease	59 (1.5)	760 (0.7)
Mild liver disease	33 (0.8)	534 (0.5)
Diabetes	321 (8.2)	5655 (5.6)
Diabetes with end organ damage	141 (3.6)	1884 (1.9)
Hemiplegia	14 (0.4)	127 (0.1)
Moderate or severe renal disease	117 (3.0)	1710 (1.7)
Any tumor, leukemia, lymphoma	454 (11.6)	10,162 (10.0)
Moderate or severe liver disease	18 (0.5)	132 (0.1)
Metastatic solid tumor	121 (3.1)	2262 (2.2)
Other drug use (*N*, *%*)		
Blood glucose lowering drugs, excluded insulins	306 (7.8)	6982 (6.9)
Hormone replacement therapy	759 (19.5)	25,612 (25.2)
Immunosuppressant drugs	185 (4.7)	3738 (3.7)
Insulin and analogues	149 (3.8)	2326 (2.3)
Proton pump inhibitor	1252 (32.1)	29,001 (28.6)
Reimbursement according to NorPD (ICPC code^c^ or ICD-10 code^d^) (*N*, *%*)		
Rheumatoid arthritis	342 (8.8)	8699 (8.6)
Other inflammatory rheumatic disease	269 (6.9)	6543 (6.4)
Other indication	772 (19.8)	17,439 (17.2)
Indication not known	214 (5.5)	4702 (4.6)
Indication according to NPR^e^ (*N*, %)		
Rheumatoid arthritis	32 (0.8)	470 (0.5)
Other inflammatory rheumatic disease	211 (5.4)	2124 (2.1)
Other indication	365 (9.4)	6762 (6.7)

*IQR* interquartile range, *DDD* Defined Daily Dose, *CCI* Romano modification of the Charlson comorbidity index

Women already treated with anti-osteoporotic drugs when starting GC were excluded (5 years look-back)

^a^According to Charlson comorbidity index (CCI), Romano modification

^b^Connective tissue disease is the term used in CCI, commonly used term is rheumatic inflammatory diseases

^c^Reimbursement code associated with first dispensing of GC (primary care). International Classification of Primary Care (ICPC)

^d^Reimbursement code associated with first dispensation of GC (specialist health care). International Statistical Classification of Diseases and Related Health Problems, 10th revision (ICD-10)

^e^Patient with indication according to the NPR (and no indication in NorPD at first GC dispensing) in the 2-year baseline period. If more than one relevant diagnosis, the one closest in time before first GC dispensing was chosen

Women with and without previous fracture had similar amount of reimbursement at their first GC dispensing, 41% versus 37%, respectively. Among those with reimbursement, the majority had a known reimbursement code (known indication) (Table 1).

A total of 4504 women started AOD during follow-up (minimum 1 year and maximum almost 8 years), where 3256 women had started AOD and 79,638 were censored after 1-year follow-up. Among women with rheumatoid arthritis or other inflammatory rheumatic disease, 50% had discontinued GC treatment after 1-year follow-up versus 80% among women with other GC indications and 86% with no known GC indication. Among women who started AOD treatment (and without a GC treatment gap of more than 180 days), median time between the first GC and the first AOD prescription dispensation was 592 days (interquartile range (IQR): 203, 1209) for women without a previous fracture and 417 days (IQR: 149, 944) among women with a previous fracture.

**Fig. 2:**
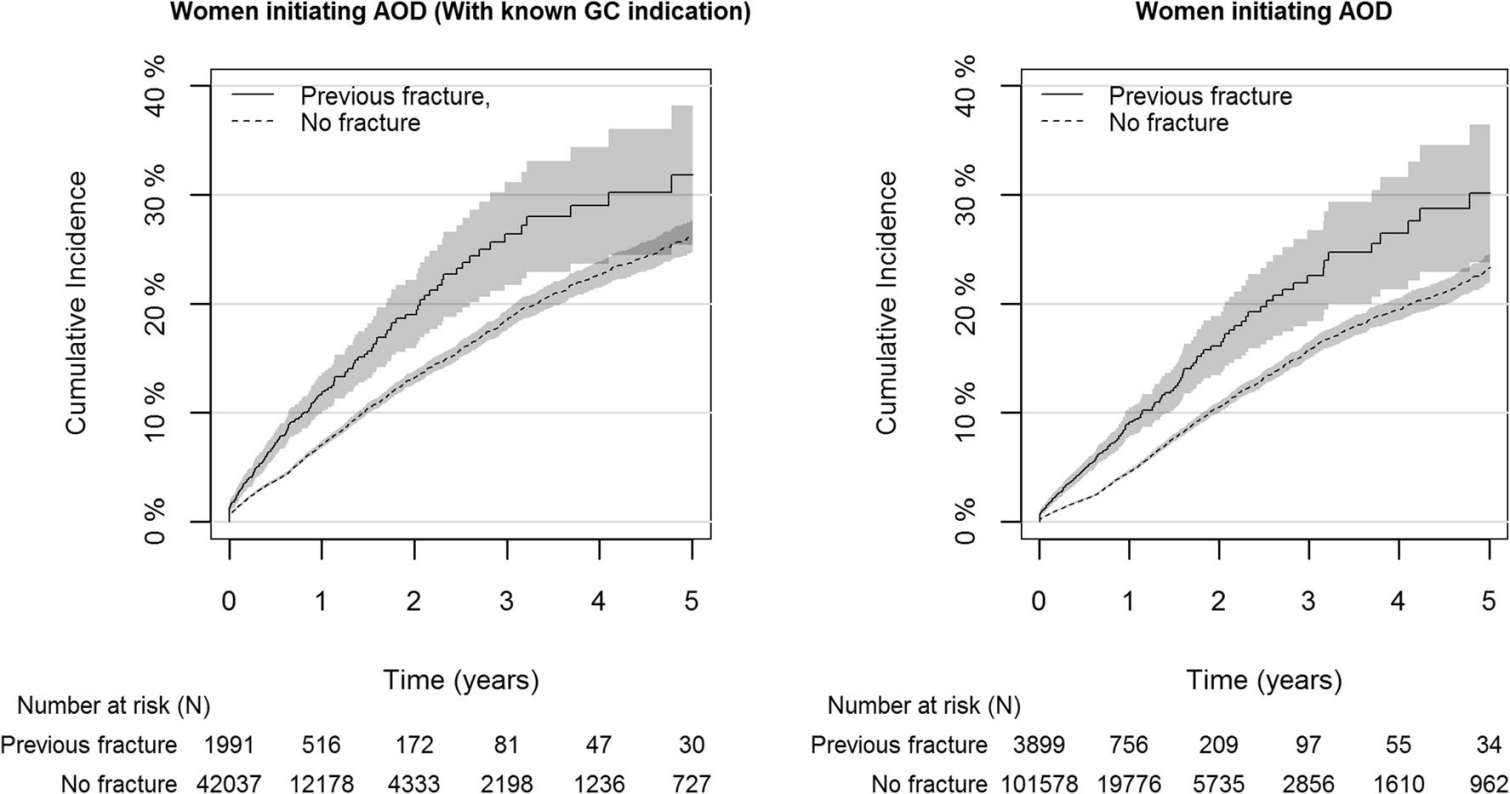
Cumulative incidence (with 95% confidence bands) of women starting treatment with anti-osteoporotic drugs (AOD) after initiation of glucocorticoid (GC) treatment 2010–2016, stratified by having had a previous fracture or not during the previous 2 past years. The figure to the left shows data for women with a known chronic disease requiring long-term treatment (*N* = 44,028). The figure to the right includes all women starting GC treatment (*N* = 105,477)

Cumulative incidence of starting AOD was higher at any time point among women with versus without a previous fracture. This was true both for women with known GC indication and among all women (Fig. 2). Further, cumulative incidence of starting AOD at 1-year follow-up was slightly higher among women with a known GC indication compared with all women, both for women with previous fracture and no fracture, and this difference persisted through the 5-year follow-up (Fig. 2). Among all women initiating GC, the cumulative incidence of starting AOD within 1 year was 9.1% (95% CI: 7.9, 10.4) among women with fracture versus 4.6% (95% CI: 4.4%, 4.8%) among those without fracture (Fig. 2). After 5-year follow-up, cumulative incidence was 30.2% (95% CI: 23.8%, 36.5%) among women with fracture versus 23.2 (95% CI: 21.9%, 24.5%) among women without.

Among women with known indication for GC treatment, the cumulative incidences at 1-year follow-up were 11.8% (95% CI: 10.0, 13.5) for women with a fracture and 7.1% (95% CI: 6.8, 7.4) without fracture (Fig. 2).

**Fig. 3:**
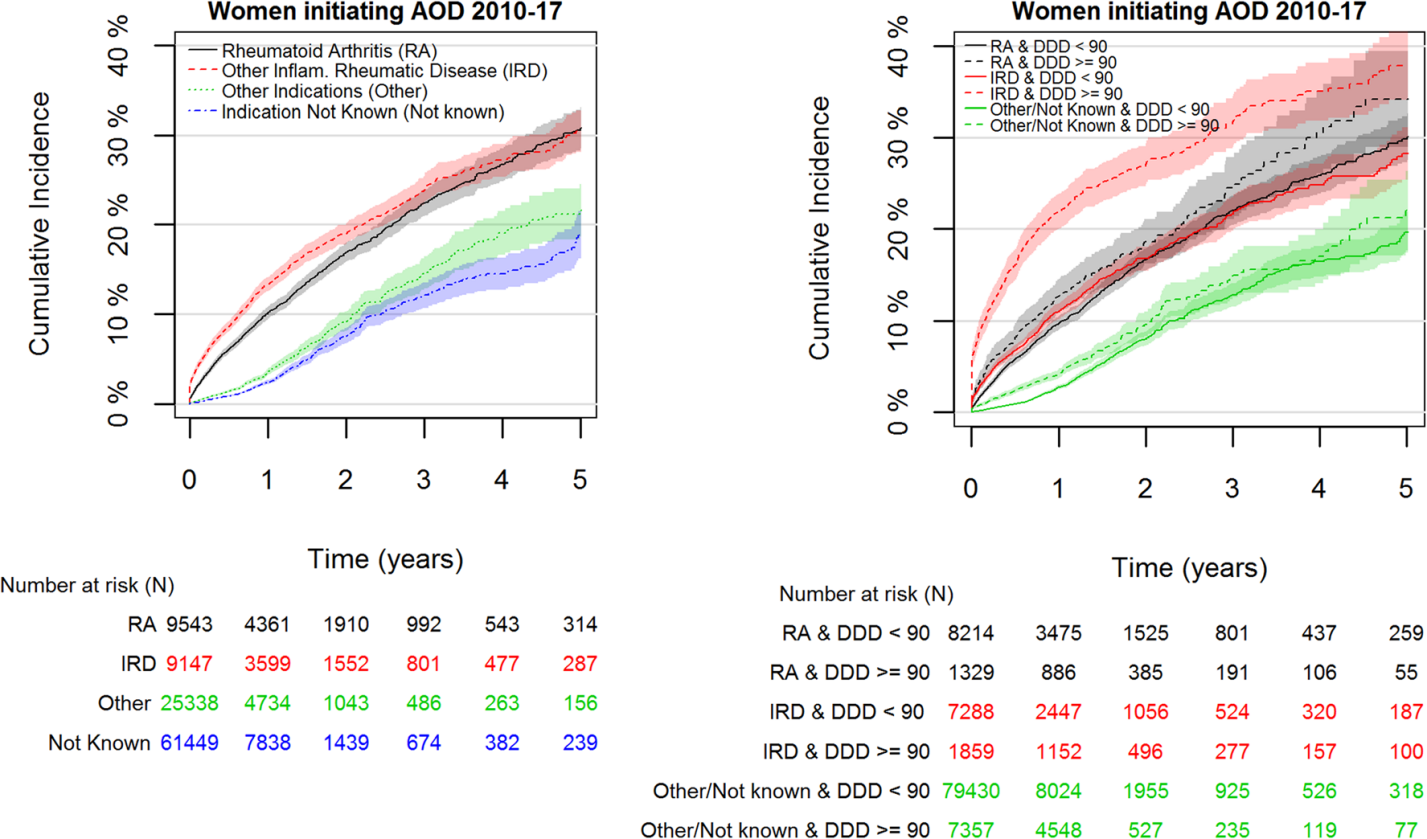
Cumulative incidence (with 95% confidence bands) of women 55–84 years in Norway of starting treatment with anti-osteoporotic drugs (AOD) after initiation of glucocorticoid (GC) treatment 2010–2016, stratified by GC indication (left) and by initial Defined Daily Dose of GC and GC indication (right)

Cumulative incidences of receiving AOD within 1 year after initiation of GC treatment among women with rheumatoid arthritis and other inflammatory rheumatic diseases were 10.1% (95% CI: 9,5, 10.8) and 13.3% (95% CI: 12.5, 14.1), respectively. This was 2–3 times higher (and decreasing over time) than for women with other indications and for indication not known (cumulative incidence 3.6 (95% CI: 3.2, 3.9) and 2.4 (95% CI: 2.2, 2.7), respectively) (Fig. 3, left). After 5-year follow-up, the cumulative incidences were 30.6% for rheumatoid arthritis, 30.4% for other inflammatory rheumatic disease, 21.6% for other indications, and 18.8% for indication not known.

Cumulative incidence of receiving AOD within 1 year after initiation of GC treatment was higher among women receiving an initial dose of GC of more than 90 DDD for any indication, and this difference was highest among women with other inflammatory rheumatic diseases (Fig.3, right). A majority of women received an initial dose of less than 90 DDD, and a high proportion of these continued GC treatment beyond 1 year.

The higher 1-year probability of receiving AOD in the fracture group persisted after adjusting for baseline covariates (age, education, and year of initiation of GC), with a HR of 2.0 (95% CI: 1.8, 2.3) (Table 2). Among women with rheumatoid arthritis or another inflammatory rheumatic disease as indication for GC treatment, the probability of starting AOD within 1 year was 1.5–1.6 times higher when having had a previous fracture versus no fracture (Table 2). Among women with other known indications for GC treatment and for women with no known indication for GC treatment, the difference between the fracture and no fracture group was 2.4–2.6-fold (Table 2). Stratifying on initial dose of GC gave slightly higher HR for DDD < 90 compared with DDD > = 90 for fracture vs. no fracture group (Table 2).

**Table 2:** Age-standardized incidence rates and risk (HR) of initiating anti-osteoporotic drugs (previous fracture vs. no previous fracture) in 105,477 Norwegian women (55–84 years old) initiating glucocorticoid (GC) treatment in the period 2010–2016

	No previous fracture		Previous fracture		
	*N*/person years	Adj. IR^1^	*N*/person years	Adj. IR^1^	Adj. HR^2^ (95% CI)
All women initiating GC	3004/70,264	4.2 (4.1, 4.4)	257/2645	9.0 (7.9, 10.3)	2.0 (1.8, 2.3)
Indication for GC^3^					
Rheumatoid arthritis	788/7245	10.3 (9.5, 11.1)	53/283	16.3 (11.2, 24.4)	1.6 (1.2, 2.1)
Other inflammatory rheumatic disease	981/6471	14.4 (13.5, 15.4)	79/331	21.8 (16.9, 28.0)	1.5 (1.2, 1.9)
Other Indications	491/16,804	2.9 (2.7, 3.2)	59/774	7.8 (5.8, 10.3)	2.4 (1.8, 3.2)
Indication not known	744/39,744	1.9 (1.8, 2.0)	66/1257	5.1 (3.9, 6.6)	2.6 (2.0, 3.4)
Number of Defined Daily Doses dispensed of GC at initiation of GC					
< 90 DDD	2208/61,495	3.5 (3.4, 3.7)	198/2297	7.7 (6.6, 9.0)	2.2 (1.9, 2.5)
> = 90 DDD	796/8769	8.9 (8.3, 9.5)	59/347	18.3 (13.7, 24.2)	1.7 (1.3, 2.2)

*HR* hazard ratio, *IR* incidence rates, *DDD* Defined Daily Doses

All women initiating GC by indication for GC use and by number of Defined Daily Doses of GC dispensed at initiation of GC 1-year follow-up

^1^Age-standardized incidence rates per 100 person years

^2 ^“No previous fracture” as reference. Adjusted for age at initiation of GC, index year, and education

^3^Based on reimbursement codes at first GC dispensing (NorPD) or on diagnostics codes (ICD-10) in the 2-year baseline period (NPR)

**Fig. 4:**
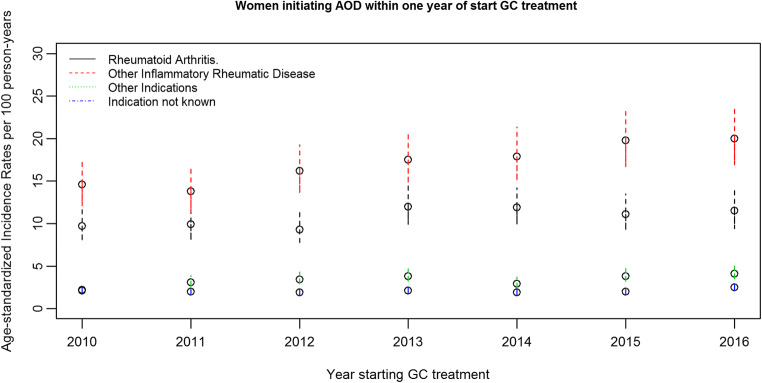
Age-standardized incidence rates with 95% confidence intervals of starting with anti-osteoporotic drugs (AOD) within 1 year after initiating glucocorticoid (GC) treatment by type of GC indication, as a function of year of initiating GC treatment

Age-standardized incidence rates of starting AOD within 1 year after initiating GC showed an increasing trend in the period 2010–2016 for all studied GC indications (Fig.4). Adjusting for age and education, the 1-year probability of receiving AOD, among all women initiating GC, increased on average 3% per year (HR = 1.03, CI: 1.01, 1.05) from 2010 to 2016. For those with rheumatoid arthritis as indication for GC treatment, the yearly increase was 3% (HR = 1.03, CI: 1.00, 1.07); for other inflammatory rheumatic diseases, the increase was 4% (HR = 1.04, CI: 1.01, 1.07), for other indications 7% (HR = 1.07, CI: 1.02, 1.11), and for no known indication 3% (HR = 1.05; 1.03, 1.07). Among women with previous fracture, the increase was 10% per year (HR = 1.10, CI: 1.03, 1.17). There was no significant interaction between these time trends and type of GC indication or with fracture status.

### Sensitivity analysis

Repeating the analyses reducing the grace period from 180 to 90 days before censoring patients yielded slightly lower HRs for receiving AOD at 1-year follow-up for women with fracture vs. no fracture (Table 2, 180 days). Cumulative incidences of starting AOD within 1 year increased from 10.1 to 11.6% for rheumatoid arthritis, from 13.1 to 15.4% for other inflammatory rheumatic diseases, from 3.6 to 5.3% for other indications, and from 2.4 to 4.2% for indication not known. The corresponding cumulative incidences at 5-year follow-up were 37%, 36%, 27%, and 26% for these GC indications, respectively.

## Discussion

### Summary

Among postmenopausal women starting GC therapy, those who had suffered a fracture during the previous 2 years were twice as likely to start treatment with AOD than women without fracture, and this association was strongest among women with no known indication for GC treatment (HR = 2.6) and in the group “other GC indication” (chronic obstructive lung disease, asthma, sarcoidosis, dermatitis, and ulcerative colitis) (HR = 2.4). Overall, women with a diagnosis of inflammatory rheumatic disease were 2–3 times more likely to start treatment with AOD within the first year compared with women without this condition. Further, women given an initial dose of GC of more than 90 DDD were more than twice as likely to start AOD treatment within 1 year than women given less than 90 DDD, both for women with and without previous fracture. However, the vast majority of women on GC treatment were initially prescribed less than 90 DDD, and a large proportion of these continued GC treatment beyond 1 year, which may suggest that AOD treatment should have been initiated. During the years 2010 to 2016, the rate of AOD prescribed to those initiating GC increased on average 3% per year, and this positive trend was higher among women with (10%) versus without (3%) previous fracture, as well as among women with other indications for GC treatment (7%), compared with women with inflammatory rheumatic disease (3–4%).

### Earlier fracture and fracture risk/undertreatment

According to both previous and current guidelines, patients with previous fractures as well as those receiving GC therapy for more than 3 months are at a high risk for fractures, and AOD should be considered [10, 12]. The present study found that women with a recent fracture who initiated GC treatment subsequently started AOD treatment almost twice as often as those without a previous fracture. Women in the fracture cohort were older and had more comorbidity than those without recent fractures; thus, the proportion starting AOD should be high. However, the AOD treatment rates were low, even in this group. It is worth noticing that among women starting AOD, the median time from starting GC to prescribing AOD was more than 1 year, both among those with and without previous fractures. Low treatment rates in high-risk patients have also been reported in a Danish study, where only 1/3 of the hip fracture patients started AOD during the subsequent year [36].

Reimbursement rules have been important for choice of AOD and decisions concerning whom to treat. In Norway, osteoporosis confirmed by a BMD measurement was required between 2006 and 2011 , excluding those living in rural areas without any available device for BMD measurements. In addition, up to 2012, AOD were only fully reimbursed after a fracture [15]. Thus, women without a previous fracture or who did not have a BMD-verified osteoporosis diagnosis had to pay the full costs for the AOD treatment. This may explain the low AOD treatment rate in women initiating GC without a previous fracture. Reimbursement for corticosteroid-induced osteoporosis was introduced in 2013.

In our nationwide cohort, around 10% of women with a recent fracture were on AOD after 1 year (incident use) from GC initiation. In a community study in Central Norway among women 50–85 years old with a > 20% estimated 10-year risk of sustaining a major osteoporotic fracture, only 25% were treated with AOD, further illustrating the level of undertreatment [37]. There is, however, increased attention to some risk factors, and long-term use of GC in combination with sustaining a hip fracture was the strongest predictor for starting AOD in another Norwegian study [38].

A Canadian study found that women who started taking GC and who had a previous fracture were more often admitted to BMD measurements or to receive AOD than those without a fracture [39]. A French study reported that 26% of women above 55 years starting GC were treated with osteoporosis medication, which also included those who only started supplements of calcium and vitamin D [40]. Thus, the proportion on AOD was even lower.

### Inflammatory rheumatic diseases

In our study, women with a diagnosis of an inflammatory rheumatic disease were more likely to start AOD treatment within 1 year from GC initiation, compared with women with other diagnoses, even without having had a recent fracture. Studies from Norway, the USA, and France have found that rheumatologists refer patients on GC treatment to BMD measurements and start AOD more frequently than other specialists [38, 40, 41]. The American College of Rheumatology published guidelines for prevention and treatment of GC-induced osteoporosis as early as in 1996, and the awareness about inflammation, as well as GC, as a contributing factor to osteoporosis is high among rheumatologists [42–45]. Equipment for measuring BMD is often allocated to rheumatologic practices, which lowers the threshold for identifying those at risk and starting osteoporosis prophylaxis and treatment. Despite this, a recent French study investigating whether the guidelines for prevention of GC-induced osteoporosis were applied in patients with RA found that less than 30% of those with an indication for treatment received AOD [46]. Guidelines may not be sufficient, and a Japanese group decided to increase education and attention to GC-induced osteoporosis at their hospital by introducing a quality indicator to monitor prevention and treatment of GC-induced osteoporosis [47]. They found that these interventions improved the proportion of patients treated with AOD during 2010–2013.

Only a few women with SLE were included in the present study, and none of these was registered with a fracture. The incidence of SLE is low in this age group, and thereby few start GC treatment—thus, most women 55 years and older with SLE had already started using GC when they were younger and were therefore not eligible for inclusion (i.e. they were not incident users). In a Spanish study including 576 women with RA or SLE using long-term GC, 19% had radiologically confirmed vertebral fracture. The prevalent use of AOD was about 50% for the total cohort and about 80% for those with a radiologically confirmed vertebral fracture [48]. This may indicate that many patients with rheumatic disease on GC start AOD treatment eventually, but preventive treatment may be delayed.

Most patients with PMR/GCA are treated with GC for several months or years, and AOD would be indicated. The majority of patients with inflammatory disease other than RA in our study had PMR/GCA, and this group had the highest HR for starting AOD treatment (adjusted for age). In a Danish study, only half of the cohort with PMR/GCA were treated with AOD even though this is a high-risk population with median age 73 years and a majority of women [49].

### Trends

We found that AOD was increasingly dispensed to women initiating GC during the years 2010 to 2016, especially from 2013 when bisphosphonates were reimbursed for corticosteroid-induced osteoporosis. However, this reimbursement was only for 10 mg alendronate in daily oral administration, not for the much more frequently prescribed weekly administration. In an earlier Norwegian study, a decrease in the initiation of AOD was found during 2004–2007 for a population above 40 years [17]. In the previously mentioned study from Central Norway, the rate of initiation of AOD after a forearm fracture did not change between 2005 and 2012 [15]. Our finding is consistent with an increased awareness of the negative effects of GC on the bone as the AOD treatment rate increased over time in GC starters. Our finding is in concordance with the Canadian study reporting a 51% increase in GC-induced osteoporosis preventive care (including both BMD testing and AOD prescribing) in new users of GC during the years 1998–2008 [39].

### Limitations/strengths

The strength of this study is the nationwide cohort of all Norwegian women 55–85 years old and the long follow-up time. We aimed to describe to which degree AOD was dispensed to women initiating GC therapy, thereby focusing more on the attentiveness of the physicians to anti-osteoporotic treatment rather than the patients’ adherence to treatment. As in all studies based on pharmacy-dispensed medications, the actual number prescribed maybe higher than the number dispensed, in which case the dispensing rates will underestimate the actual prescribing rates. Further, we cannot know whether the medication dispensed was actually taken. Some information on the use of intravenously administered bisphosphonates may be lacking, as medications administered in hospitals and nursing homes are not registered in the NorPD. Using hospital data on intravenously administered bisphosphonates (Zoledronic acid) and indication (excluding cancer diagnosis), both obtained from the NPR, suggests that the use of AOD is underestimated with about 5%.

We also lack information about treatment with AOD before the look-back period of 5 year, and some of the patients may have been on a “drug holiday” after long-term treatment with AOD and not considered eligible for starting AOD again. The patient’s motivation is also important for AOD treatment; the treating physician may have considered AOD and decided not to start due to comorbidity or patient’s resistance to therapy. There may be some slight under-reporting of fractures, as only fractures registered in hospital in- and outpatient clinics and emergency departments, and not those treated only by the general practitioner, are included in the Norwegian Patient Registry.

As 14.5% of those without a fracture in our study population were on HRT before initiating GC, this may have been one reason for not starting AOD. HRT has been found to be a negative predictor for starting AOD in an earlier study [15].

Bone mineral density data are not available in the national registry data such as those used in our study, and this represents a limitation. At the population level, the majority of women in this age group have either osteoporosis or osteopenia and should therefore be eligible for osteoporosis prophylaxis when starting GC treatment had bone mineral density been measured.

We used 180 days as the allowable treatment gap before censoring GC patients. Therefore, the number still on GC treatment and at risk of starting AOD the first year after treatment may be artificially high, compared with if we had used a smaller treatment gap, such as 90 days. On the other hand, when using 180 days as the allowable treatment gap, we increased the number at risk 2–4 times, depending on GC indication and follow-up time, compared with using 90 days as the treatment gap.

## Conclusion

Although postmenopausal women starting long-term GC treatment are at a high risk of fracture, the majority did not start treatment with AOD following GC initiation. The probability of starting AOD was especially low for women initiating GC with no reported indication for this treatment. However, we did observe a slight increase in AOD, within 1-year treatment rates, for women initiating GC from 2010 to 2016 and especially from 2013 when AOD reimbursement for corticosteroid-induced osteoporosis became available. Women with a previous fracture or who had an inflammatory rheumatic disease were more frequently treated with AOD after initiating GC, but even in these groups the recommendations in guidelines for osteoporosis prophylaxis were often not followed. The bone harming effect of GC may not be fully appreciated by physicians, and better routines for prevention of GC-induced osteoporosis and fractures should be established. Further, reimbursement rules should better reflect the international guidelines not only for osteoporosis treatment but also for prophylaxis.

## Electronic supplementary material

ESM 1 (DOCX 18 kb).
