# Supplementary material for: Initiation of anti-osteoporotic drugs in high-risk female patients starting glucocorticoid treatment: a population study in Norway
Source: Arch Osteoporos. 2020 Aug 5;15(1):121. doi: 10.1007/s11657-020-00783-8 (PMC7406535; doi:10.1007/s11657-020-00783-8)
Supplement: Supplementary file 1 — (DOCX 18 kb). [file 11657_2020_783_MOESM1_ESM.docx]

Supplementary (web material)

**Table S1**. Glucocorticosteroids^1,2^ and Antiosteoporotic^1,3^ drug included in the study. Data from the Norwegian Prescription Database (NorPD).

| **Drug group** | **Drug name** | **ATC-code^4^** |
| --- | --- | --- |
| Glucocorticosteroids (GC) | Prednisolone | H02AB06 |
| Antiosteoporotic Drug (AOD) | Alendronate, Risedronate, Zoledronic acid (Aclasta), Etidronate, Denosumab, Teriparatide, Raloxifene | M05BA04, M05BA07-08, M05BB01, M05BX04, H05AA, G03XC01 |
| ^1^Data from NorPD 2005-2017.  ^2^GC commonly used as glucocorticoid substitution treatment (cortisone) was not included, neither was GC for intravenous, intramuscular or intra-articular injections, nor for topical treatment.  ^3^Calcium supplements up to 1500 mg and vitamin D3 up to 20 µg are available without prescription in Norway and were therefore not used to define AOD.  ^4^In Norway, drugs are classified using the Anatomical Therapeutic Chemical (ATC) classification system (<https://www.whocc.no/>). | | |

**Table S2.** Fracture diagnoses included in the study; Data from the Norwegian Patient Registry (NPR)^1^.

| **Type of fracture** | **Diagnostic codes (ICD-10**^2^**)** |
| --- | --- |
| Hip | S72.x |
| Pelvis | S52.x, S22x, T08.x |
| Spine | S32x |
| Femur/Humerus | S42.x |

^1^ Norwegian Patient Registry (NPR) 2008-2017.

^2^International Classification of Diseases, 10^th^ version

**Table S3**. Glucocorticosteroids reimbursement codes included in the study from Specialist Health Care and Primary Health Care using data from the Norwegian Prescription Database (NorPD).

| **Exposure group** | **Reimbursement codes from Specialist Health Care (ICD-10)** | **Reimbursement codes from Primary Health Care (ICPC2)^1^** |
| --- | --- | --- |
| Rheumatoid Arthritis | M05-06 | L88 |
| Other Inflammatory Rheumatic Disease | All M-codes except M05-06 | L99 |
| Other Indication | D59, D69, D81-D84, D86,E23-E27,E89,G70, H20,H30,I67, J30, J43-45, J84, K20,K50-52,K72, K75,L20-23, L30,L40,L98,N01-N03, Z94 | A87, B82-83, B99,D84,D94,D97,F73,R95-R99,S86-S91,S99,T99,  U88, |

^1^Data from NorPD 2005-2017.

^1^**International Classification of Primary Care (ICPC)**

^2^International Classification of Diseases, 10^th^ version

**Table S4**. Indication for Glucocorticosteroids treatment; Data from Norwegian Patient Registry NPR)^1^.

| **Indication (Exposure group)** | **Diagnostic code (ICD-10**^2^**)** |
| --- | --- |
| Rheumatoid arthritis | M05.x, M06.x |
| Other rheumatic inflammatory disease | All M-codes except M05.x and M06.x |
| Other Indication: chronic obstructive lung disease, asthma, sarcoidosis, dermatitis, ulcerative colitis | J43.x-45.x,J84.x,K50.x-52.x,K75.x,Z94.x,L20.x-23.x,L30.x,L40.x,L98.x,D84.x,D86.x. |
| ^1^ Norwegian Patient Registry (NPR) 2008-2017..  ^2^International Classification of Diseases, 10^th^ version | |
